# Relationship between lifestyle pattern and blood pressure - Iranian national survey

**DOI:** 10.1038/s41598-019-51309-3

**Published:** 2019-10-23

**Authors:** Samaneh Akbarpour, Davood Khalili, Hojjat Zeraati, Mohammad Ali Mansournia, Azra Ramezankhani, Mahin Ahmadi Pishkuhi, Soroush Rostami Gooran, Akbar Fotouhi

**Affiliations:** 1 Occupational Sleep Research Center, Baharloo Hospital, Tehran University of Medical Sciences, Tehran, Iran; 2 Prevention of Metabolic Disorders Research Center, Research Institute for Endocrine Sciences, Shahid Beheshti University of Medical Sciences, Tehran, Iran; 3 Department of Biostatistics and Epidemiology, Research Institute for Endocrine Sciences, Shahid Beheshti University of Medical Sciences, Tehran, Iran; 4 Pars Advanced and Minimally Invasive Medical Manners Research Center, Pars Hospital, Iran University of Medical Sciences, Tehran, Iran; 5 Faculty of computer Engineering, Shaid Rajaee Teacher Training University (SRTTU), Tehran, Iran; 6 Department of Epidemiology and Biostatistics, School of Public Health, Tehran University of Medical Sciences, Tehran, Iran

**Keywords:** Epidemiology, Health occupations

## Abstract

We aimed to evaluate the relationship between different lifestyle patterns and blood pressure. This study is based on the national survey of the risk factors for non-communicable diseases in Iran in 2012. A total of 8244 people aged 25–70 years old have been enrolled in the survey. Clustering on the individual data of lifestyle factors (nutrition, physical activity, and smoking) were carried out using self-organizing neural network method. Multivariable regression models were used to determine the relationship between blood pressure and the clusters. This study revealed seven lifestyle clusters in the national survey. The first cluster had a healthier lifestyle (15%), and the rest of the clusters had at least one or more lifestyle-related risk factors. Among all the clusters, people in two clusters, i.e. one characterized by consumption of sugar-sweetened beverages, salt, and fast foods, and the other one characterized by physical inactivity, were more exposed to the risk of hypertension (odds ratios of 1.44 and 1.12, respectively). People in another cluster who were 100% smokers and had a very high level of work-related physical activity were about 30% less likely to experience elevated blood pressure. Although a lifestyle with cigarette smoking was associated with a reduction in blood pressure, this might be due to other related factors, such as work-related physical activity, which lower blood pressure. Of course, this hypothesis still needs to be further studied in the future.

## Introduction

Elevated blood pressure is one of the most important health problems, which is considered as one of the major risk factors for cardiovascular disease, stroke, and kidney diseases as well^1,2^. Various studies have shown that the prevalence of hypertension is increasing in various countries^3–5^, however, the highest level of increase has been observed in developing countries, especially in Asia and the East Mediterranean region. According to the Iranian Health Profile Survey, around 22% of the adult population are hypertensive and the problem is more prevalent in the population above 55 years old. The maximum and minimum rate of hypertension within Iran is in Tehran and Qazvin with the prevalence of 47% and 6.9%, respectively. In Iran, the prevalence of hypertension is higher in urban areas^6^.

According to many studies, this increase is due to changes in lifestyle and an increase in behavioral risk factors such as unhealthy nutrition, physical inactivity, and smoking^7–10^.

So far, many different studies have separately examined the relationship between different lifestyle-related factors and blood pressure, but these studies have neglected the fact that lifestyle-related risk factors usually occur together in a person and tend to be clustered.

The clustering of health-related behavioral risk factors is a major threat to the public health. Recent studies have shown that the coincidence of health-related risk factors in a person can result in synergistic effects that in turn increase the risk of non-communicable diseases and subsequent mortality^11,12^. A cohort study that was conducted on 20,244 Americans after 11 years of follow-up showed that people with all the four major risk factors to health (cigarette smoking, alcohol consumption, physical inactivity, and poor nutrition), compared with those without any of these four risk factors, were four times more prone to death^12^. Other studies in European and American countries have reported similar patterns on the association between mortality and these risk factors^13,14^.

Very few studies have been conducted and published on the relationship between different lifestyle clusters and elevated blood pressure^15^. For example, in a study conducted by Thani *et al*., it was shown that there were three lifestyle patterns in Qatar, and people in clusters characterized by specific characteristics such as smoking, physical inactivity, and fast food consumption, were two times more prone to the risk of developing high blood pressure, as compared with those who had a healthy lifestyle^15^.

Therefore, it seems that blood pressure varies among people in different lifestyle groups. Understanding the characteristics of the various lifestyle subgroups (clusters) and their relationships with blood pressure can help identify different subgroups at a high risk for hypertension and plan appropriate interventions for them.

Although many different studies have been conducted in the Iran assessing the association between different individual lifestyle-related variables and blood pressure^16–18^, so far to the best of our knowledge, no study has examined the relationship between the clustering of lifestyle risk factors and blood pressure at the national level. *self*-*organizing* map (SOM) is an artificial neural network clustering technique that can be used for grouping similar individuals that has been introduced since 1982^19,20^. Although there are several limitations for mapping data points and other data-mining methods are needed to be used in combination with this method to overcome this limitation, SOM provides the great advantage of identifying clusters and their sub-clusters on the maps and makes the complex algorithms easily understandable^21^. Thus, this study was carried out with SOM technique to achieve the goal.

## Methods

### Study population

The present study was conducted on data obtained from the national study of major risk factors for non-communicable diseases in 2012. The protocol for the national survey of risk factors of non-communicable disease (SuRFNCD) of Iran was approved by the Iran’s Center for Disease Control (CDC). For all the procedures performed in this study, verbal informed consent was obtained from all individual participants in the study, and for study participants under the age of 18 years verbal informed consent was obtained from their parents and/or a legal guardian. Details of this study have been reported elsewhere^22^. In brief, the mentioned survey was carried out on 12,000 people aged 6 to 70 years old through the guidelines and regulations recommended by the World Health Organization. This study first used stratified sampling (the strata included provinces, cities, or villages) and then utilized cluster sampling (within each strata). Given that people of very high and very low age groups are expected to have different lifestyles and blood pressure, so the study population was limited to people between the ages of 25 and 70. Finally, after cleaning the data, a total of 8244 people were enrolled into the present study.

To collect data, we used a standard questionnaire that was prepared based on the WHO’s guidelines for the stepwise study of non-communicable disease risk factors. The collected data included demographic data (age, sex, education, and occupation) and information about the lifestyle-related behaviors such as nutrition (7 variables including; mean servings of fruit, vegetables, and dairy products in a day, mean number of days per week for fast food and sweet soft drinks, adding salt to food, and predominant use of unsaturated fat vs. saturated fats for cooking), physical activity (3 variables including; hours a week for work related or recreational physical activities, and walking), smoking (2 variables including; cigarettes and hookah consumption), and alcohol consumption. The methods of measuring variables and reviewing the patterns of health-related behaviors were presented in the previous report^23^.

In order to measure the blood pressure of the subjects, they were asked to sit down calmly for five minutes. Then, blood pressure was measured at the right arm. Blood pressure measurements were repeated in two consecutive rounds, and five minutes of rest between each round was recommended. The mean value of the two measurements was considered as the blood pressure. People with a systolic blood pressure (SBP) ≥140 mmHg or with a diastolic blood pressure (DBP) ≥90 mmHg and those taking blood pressure lowering medications were considered as patients with high blood pressure^24^.

### Statistical analysis

In order to describe the quantitative variables, we reported mean and standard deviations (SD), and to describe qualitative variables the frequency and percentage were reported. Approximately, 3.23% of the data cells had not been recorded. Therefore, the missing (unknown) values were imputed by the single imputation method and regression model in mice package using R software^25,26^. A *self*-*organizing* map (SOM), a type of artificial neural network (ANN), was used for clustering of lifestyle risk factors^19,20^. Further details about this method have been presented elsewhere^20^. Details of the lifestyle factors in each of the clusters was described in two tables in appendix that were reported in our previously published paper^23^.

In order to study the relationship between blood pressure and lifestyle patterns, blood pressure was defined in three ways. First, SBP and DBP were evaluated separately in the linear regression model, then high blood pressure was defined as a bivariate variable and their relationship were evaluated in a logistic regression model. When studying the relationship between high blood pressure and lifestyle patterns in the logistic model, age, sex, job, and education were studied as confounding variables. In linear regression model, which was used to study the relationship between systolic and diastolic blood pressure and lifestyle patterns, in addition to the mentioned variables, the use of hypertension drugs was also assessed. It is worth noting that, the standard population of the country was used for weighing in the survey analysis.

### Information about previous presentations of the whole or part of the work presented in the article

In the present study we used the known pattern (clusters) of lifestyle risk factors in Iran that was reported in last paper. Last paper was accepted in Caspian internal medicine journal and is in press. We reported it in the 19 references.

## Results

A total of 8244 people were enrolled into the study, with a mean age of 42.12 years and a SD of 13.60 years. Of all, 3305 subjects were male. Table 1 presents the status of participants in terms of other demographic variables such as occupation and education.

**Table 1:** Sociodemographic characteristics of participants.

Variables	Mean	95% CI
Age (year)	42.21	422.42–14.28
	**Frequency**	**Percent**
Sex (male)	3305	49.63
Residence (urban)	5798	69.44
Education category		
Under diploma	5637	62.57
diploma	1572	22.02
Upper diploma	1035	15.41
Job category		
Employee working in office	647	10.36
Workers in factory environment	542	8.27
Self-employed	1633	25.14
Housewife	4062	40.10
Others	1360	16.13

Age is reported as with weighted mean (95% Confidence interval) and others are reported with frequency (unweighted) and weighted percent (Data were weighted based on the 2011 national Iranian population aged ≥25 and ≤70 years).

The mean consumption of fruits, vegetables, and dairy products, respectively, was 1.17, 0.92, and 1.65 units per day. The mean number of days in which the participants consumed fast food and sugar-sweetened beverages was 0.38 and 1.64 days per week, respectively. About 54.5% of the subjects consumed salt with foods and 41.7% used predominant use of unsaturated fat. The studied participants reported 1.49, 0.39, and 0.59 hours of physical activity during work, recreation, and walking, respectively. Of all participants, 14.01% were smokers and 3.39% used hookah at the time of the study.

The results of clustering showed that in general, the studied population could be categorized into seven subgroups in terms of lifestyle related characteristics. The lifestyle-related features of each group are presented in Table 2. The details on the characteristics of each cluster were reported previously^23^.

**Table 2:** Specific characteristics of classes of lifestyle behaviors among the Iranian population in 2011.

Cluster number	Specific characteristics	N (percent)
Class 1	Healthy lifestyle behaviors group	1306 (15.84)
Class 2	High consumption of fast foods, salt, and sweet soft drinks	1026 (12.45)
Class 3	Unhealthy lifestyle behaviors group (with low physical activity)	2781 (33.73)
Class 4	With smoking and consumption of alcohol and sweet soft drinks	566 (6.86)
Class 5	Without any physical activity and low salt consumption	1169 (14.18)
Class 6	Unhealthy diet, low physical activity, no use of dairy products	647 (7.85)
Class 7	Smoking and work-related physical activity	749 (9.09)

The details on the characteristics of each cluster are published in the previous article (19).

Table 3 shows the status of blood pressure in the participants. As indicated in the table, the highest and lowest mean systolic and diastolic blood pressure were observed in cluster 5 and cluster 7, respectively; the highest and lowest systolic blood pressure were 141.84 and 121.54 mmHg, respectively, and the highest and lowest diastolic blood pressure were 83.39 and 77.01 mmHg, respectively. Moreover, the highest and lowest percentages of people with high blood pressure (64.43% and 23.72%) were in clusters 5 and 7, respectively.

**Table 3:** Mean of systolic and diastolic blood pressure and prevalence of hypertension status in seven classes of lifestyle behaviors among the Iranian population in 2011.

	Systolic blood pressure, (mmHg), Mean (95% CI)*	Diastolic blood pressure (mmHg), Mean (95% CI)*	Hypertension,**	Anti-hypertension drug,**
Total population	126.16 (124.68–127.64)	79.46 (78.79-3.87)	31.01	9.09
Class 1	123.47 (121.69–125.24)	78.79 (78.91–82.89)	24.87	6.35
Class 2	128.43 (124.96–130.82)	80.83 (78.60–81.05)	32.01	4.77
Class 3	123.76 (122.16–125.36)	79.45 (78.76–80.15)	27.98	7.14
Class 4	125.97 (124.07–127.88)	79.01 (77.37–80.64)	26.53	5.91
Class 5	141.84 (138.69–145.00)	83.39 (82.00–84.78)	64.43	34.75
Class 6	124.81 (122.28–127.34)	78.57 (76.62–80.52)	27.97	5.66
Class 7	121.54 (120.06–124.08)	77.01 (76.55–79.28)	23.72	5.54

*Data were reported as with weighted mean (95% Confidence interval).

**Data were reported as weighted percent.

Data were weighted based on the 2011 national Iranian population aged ≥25 and ≤70 years.

Hypertension was defined as a systolic blood pressure ≥140 mmHg or a diastolic blood pressure ≥90 mmHg or antihypertensive medication use.

Table 4 shows the results of the study of the relationship between blood pressure and patterns of health-related behaviors. As shown in the Table 4, it separately presents the results of linear regression model for systolic and diastolic blood pressure and the results of logistic regression model for high blood pressure. As shown, in all the three regression models, only in three clusters including cluster 2, 5, and 7 we observed a significant relationship between blood pressure and lifestyle patterns. This relationship was positive in cluster 2 and 5; however, in cluster 7 there was a negative relationship with all the three types of blood pressure. Accordingly, people in cluster 7 were about 30% less prone to the risk of high blood pressure, as compared with people in cluster 1 (OR = 0.71). In addition, systolic blood pressure and diastolic blood pressure in people in cluster 7 were, respectively, about 4.32 and 1.96 mmHg lower than those in people in cluster 1 (people with a healthier lifestyle).

**Table 4:** Association between lifestyle behavior patterns with systolic and diastolic blood pressure and hypertension (among the Iranian population in 2011).

Cluster number	Systolic blood pressure**	Diastolic blood pressure**	Hypertension***
	Regression coefficient (95% CI)	Regression coefficient (95% CI)	Odds ratio (95% CI)
Class 1	1	1	1
Class 2	5.12 (2.88, 7.40)*	1.28 (0.32, 2.46)*	1.44 (1.11, 1.88)*
Class 3	0.41 (−0.62, 1.45)	0.51 (−0.31, 1.19)	1.12 (0.95, 1.32)
Class 4	0.76 (−94, 2.47)	0.53 (−1.01, 2.05)	1.08 (0.79, 1.48)
Class 5	4.01 (1.65, 5.87)*	2.10 (1.04, 4.83)*	1.12 (1.01, 1.28)*
Class 6	1.01 (−1.19, 2.32)	0.08 (−0.78, 1.43)	1.06 (0.89, 1.02)
Class 7	−4.32 (−2.21, −1,01)*	−1.96 (−2.48, −1.11)*	0.71 (0.53, 0.92)*

*p-value < 0.05 consider as statistical significant.

**Linear regression models were used, Models adjusted for age, sex, education, job, antihypertensive medication, Body Mass Index.

***Logistic model was used, Model adjusted for age, sex, education, job, Body Mass Index.

Data were weighted based on the 2011 national Iranian population aged ≥25 and ≤70 years.

Hypertension was defined as a systolic blood pressure ≥140 mmHg or a diastolic blood pressure ≥ 90 mmHg or antihypertensive medication use.

## Discussion

This study investigated the relationship between different lifestyle patterns and blood pressure. The study assessed the relationship between systolic and diastolic blood pressure, as well as high systolic or diastolic blood pressure with lifestyle patterns, and the results showed that three clusters, including cluster 2, 5, and 7 were associated with developing high blood pressure. In cluster 2, a large number of people consumed salt with their foods, thus the elevated blood pressure was expected in this group. In cluster 5, people reported less added salt and more use of saturated vs. unsaturated fat in cooking, as compared with the other groups, but their physical activity was almost zero. The majority of people in this group this group of people were old and had a higher mean age. The elevated blood pressure in this cluster may be attributed to their age group. However, it is worth noting that, after adjusting the effect of the age, their impact was not removed. For example, concerning SBP, before entering the variable of age in the model, the blood pressure in cluster 5 was about 10.8 mmHg higher than that in cluster 1. Nevertheless, after entering the variable of age into the model, the difference reached 4.84 mmHg. This suggests that part of the risk of blood pressure in the elderly is associated with their lifestyle, which can be corrected through lifestyle modification.

But the most important finding in this section was the relationship between cluster 7 and high blood pressure.

Almost all of the individuals in cluster 7 were men with the highest level of work-related physical activity who were all (100%) smokers that is consistent with other studies^27^. Although this cluster had a significant relationship with blood pressure, we observed a negative (reversed) relationship in all the three analysis models; in other words, this group of people have a lower odds ratio of developing high blood pressure than other groups. Although this finding initially seems strange, after reviewing the related articles, it became clear that there were many articles that reported a negative relationship between cigarette smoking and hypertension and consider it as a paradox^28–33^. The important point is that researchers and authors of such articles still find no reason for such a relationship. An explanation for this finding is that obesity is a much more influential factor in elevating blood pressure; since people who smoke are often leaner than other people in the community (smoking can reduce people’s weight), this general fact may justify the negative relationship.

There are, of course, some studies that have shown that smoking increases the risk of high blood pressure^34–36^ and they have expressed some reasons for this observation. First, they attributed it to the toxic effects of carbon monoxide and other toxic substances in the cigarette on the walls of the veins; second, it might be due to the increase in the number of blood cells and the secondary viscosity of the blood due to exposure to carbon; third, it might be attributed to the adrenergic and sympathetic stimulations caused by nicotine and its metabolites^34–37^. As previously mentioned, there are some studies that have reported a reversed relationship and the reasons for these contradictions are still unknown.

Another interesting finding of the present study is that smokers were only in two clusters. In cluster 4 about 30% of the subjects were cigarette smokers, and in cluster 7 all the subjects i.e. 100% were smokers. There are two very distinct differences between these two groups: first, the work-related physical activity was very high in cluster 7, while it was lower in cluster 4. On the other hand, in cluster 4 in addition to smoking, the consumption of alcohol and hookah was also high. However, we were limited to assess the quantity of alcohol use. taking into account the body mass index (BMI) of people in these two groups, it can be observed that the BMI in cluster number 5 was about 24, but in cluster number 4 it was about 26. So, it might not be a very acceptable hypothesis to say that cigarette alone causes weight loss and therefore results in a decrease in blood pressure. It seems that cigarette smoking tends to be clustered with two other lifestyle risk factors, including alcohol and hookahs and sugar-sweetened beverages (which is proved in many studies). In such a condition no weight loss is observed and there is no negative relationship with high blood pressure. On the other hand, cigarette smoking also tends to be clustered with the work-related physical activity; in such a condition, not only weight loss occurs, but also there will be a synergistic effect between smoking and work-related physical activity that may reduce the weight and ultimately reduce people’s blood pressure. Another potential explanation is that the regular work-related physical activity plays a stronger role in these individuals or people in this cluster are in a lower socioeconomic status, and some other unmeasured/unreported factor related to lower socioeconomic status is affecting these results.

In groups of smokers who do not have a much work-related physical activity, there is no reduction in blood pressure, as they are likely to consume alcohol at the same time. Therefore, it might be the factor resulting in a controversy in the results of various studies that have examined the relationship between hypertension and smoking. It is worth noting that seeing such results will only appear in studies that analyze the factors in clusters; it highlights the need for further cluster analysis studies. On the other hand, as in cluster 4 only 30% of the subjects were smokers, therefore, further studies are needed to prove this hypothesis.

To confirm the findings, it is necessary to have two of the following conditions in the study. First, the relationship between cigarette smoking and blood pressure in cluster 4 should be a direct relationship. Second, as in our study the majority of smokers were categorized in cluster 7, this relationship should be negative in the whole population. To evaluate the first condition, the relationship between smoking and hypertension in cluster 4 was analyzed using the logistic regression model after adjusting for age and gender. The results showed that the odds ratio for cigarette smoking was 1.03 (95% CI = 0.61–1.74); although this relationship was not statistically significant, a direct relationship was observed. In order to evaluate the second condition, the relationship between smoking and blood pressure in the whole population was analyzed using the logistic regression model after adjusting all the lifestyle and demographic variables expressed in previous models. The results showed that the odds ratio was 0.61 (95% CI = 0.48–0.77), which indicates that the relation between smoking and blood pressure was negative in all the studied population. Thus, both of the mentioned conditions were met in our study.

Another point that needs to be considered when interpreting the results is that although many studies report a relationship between work-related physical activity and increased risk of non-communicable diseases, these studies have shown that the elevated blood pressure is the only non-communicable outcome that did not have a significant relationship with increased work-related physical activity. According to these studies, low level of work-related physical activity is associated with an increase in blood pressure^38^.

So far, few studies have been conducted on the relationship between non-communicable diseases and lifestyle patterns in different countries^12–14^. Moreover, a limited number of studies have examined the relationship between blood pressure and lifestyle patterns. For example, a study conducted in Qatar has examined this relationship in women^15^. This study used the main component analysis method to determine lifestyle patterns and observed three lifestyle patterns, including healthy lifestyle, unhealthy with fast food consumption and cigarette smoking, and without physical activity. The results of this study showed that the two unhealthy lifestyle patterns showed levels of risk for elevated blood pressure than the healthy one. The group that consumed fast-food and high amounts of salt at the same time showed a nearly two-fold risk of high blood pressure. These results show the mutual effect of smoking and fast food consumption. However, the present study showed that people who consumed more fast food and simultaneously consumed more salt had a higher mean level of blood pressure, but in the group that smoked and had high level of work-related physical activity, the blood pressure was much less than that in the other groups.

The common point between these two studies is the relationship between fast food consumption and high blood pressure. Many studies have shown that people who consume fast food consume higher amounts of saturated oil and salt than other people in the community, which can affect vascular function and anti-inflammatory mechanisms and ultimately increase their blood pressure^39,40^. Results of the present study are in coherence with this fact. Patients in cluster 5 had reported a higher rate of saturated fat consumption and consequently had higher risk of hypertension.

The results of the present study also showed that people who are physically inactive (cluster 5) consumed lower amounts of salt and saturated oil than other groups, however they had higher levels of blood pressure. It is worth noting that the mean age of people in this group was much higher than the mean age of people in other groups. Nevertheless, after adjusting for the effect of age and taking blood pressure medication in the regression model, this relationship was still observed. It indicates that the lifestyle of these people, which is largely associated with physical inactivity, has an independent impact on the increase in their blood pressure. In fact, it shows a synergy between physical inactivity and age. This finding is in line with the results of similar studies^41,42^. A meta-analytical study of 13 cohort studies has shown that the risk of developing hypertension has a negative relationship with a person’s physical activity^42^. Form a biological point of view, however, it has been shown that physical activity can play an important role in better vascular function and flexibility^43^. Nonetheless, no study has shown a difference between work-related physical activity and recreational physical activity in terms of their effect on the changes in blood pressure.

As one of the most important strengths of the present study, for the first time the data obtained from the national level of the country was used to identify the lifestyle patterns and its relationship with blood pressure. Cluster analysis will probably have more practical application for health policymakers, as compared with the analysis of individual factors.

As another strength of this study, we presented different definitions for work-related and recreational physical activities, as they can have a different effect on human health, while so far such a difference has not been considered by other previous studies on lifestyle patterns.

The data on the amount of consumption of two variables including salt and oil was not available. Thus adding salt to food and the type of oil, were used as substitutes and it is one of the limitations of the present study. Therefore, we only used two variables of salt intake with food and the type of oil used to determine the use of these two food ingredients. However, in most studies conducted to assess the relationship between lifestyle patterns or food patterns with blood pressure, the amount of salt intake is not measured^15,44^.

As another limitation of the present study, to collect data we relied on self-reports using a questionnaire, and we did not have a better way to measure the variables studied. However, self-reports have always their own constraints. The collected data are largely prone to underestimate the amount of smoking and alcohol consumption, which may affect the results of the present study. However, this bias in answering is observed in all questionnaires and is unavoidable.

## Conclusion

The results of this study showed a significant relationship between different lifestyle patterns and high blood pressure. People whose lifestyle is associated with salt consumption, high amounts of fast food consumption and physical inactivity are several time more prone to the risk of high blood pressure than people with a healthier lifestyle. In the elderly, although the consumption of saturated oil and salt was lower, physical inactivity regardless of the increase in age was found to be associated with higher levels of blood pressure, as compared with the other groups. This suggests that one of the most important interventions required for this group is to increase physical activity.

On the other hand, although a lifestyle with cigarette smoking was associated with a reduction in blood pressure, this might be due to other related factors, such as work-related physical activity or lower socioeconomic status which have an effect on lowering blood pressure. Of course, this hypothesis still needs to be further studied in the future.

## Supplementary information

Relationship between lifestyle pattern and blood pressure - Iranian national survey
