# Supplementary material for: Relationship between lifestyle pattern and blood pressure - Iranian national survey
Source: Sci Rep. 2019 Oct 23;9:15194. doi: 10.1038/s41598-019-51309-3 (PMC6811561; doi:10.1038/s41598-019-51309-3)
Supplement: Supplementary file 1 — Relationship between lifestyle pattern and blood pressure - Iranian national survey [file 41598_2019_51309_MOESM1_ESM.docx]

**Title:** Relationship between lifestyle pattern and blood pressure **-** Iranian national survey

**The running title:** Life style pattern and blood pressure

**Samaneh Akbarpour ^a^, Davood Khalili ^b,c^, Hojjat Zeraati ^a^, Mohammad Ali Mansournia^a^, Azra Ramezankhani^b^, Mahin Ahmadi Pishkuhi^d^, Soroush Rostami Gooran^e^, Akbar Fotouhi^a^**

^a^ Occupational Sleep Research Center, Baharloo Hospital, Tehran University of Medical Sciences, Tehran, Iran.

^b^ Prevention of Metabolic Disorders Research Center, Research Institute for Endocrine Sciences, Shahid Beheshti University of Medical Sciences, Tehran, Iran.

^c^ Department of Biostatistics and Epidemiology, Research Institute for Endocrine Sciences, Shahid Beheshti University of Medical Sciences, Tehran, Iran.

^d^ Pars Advanced and Minimally Invasive Medical Manners Research Center, Pars Hospital, Iran University of Medical Sciences, Tehran, Iran.

^e^ Faculty of computer Engineering, Shaid Rajaee Teacher Training University (SRTTU), Tehran, Iran

1. **Samaneh Akbarpour**

**Affiliation and address:** Occupational Sleep Research Center, Baharloo Hospital, Tehran University of Medical Sciences, Tehran, Iran, **Email:** [akbarpour62@yahoo.com](mailto:akbarpour62@yahoo.com)

1. **Davood Khalili**

**Affiliation and address**: Prevention of Metabolic Disorders Research Center, Research Institute for Endocrine Sciences, Shahid Beheshti University of Medical Sciences, Tehran, Iran, Department of Biostatistics and Epidemiology, Research Institute for Endocrine Sciences, Shahid Beheshti University of Medical Sciences, Tehran, Iran

**Email:** [mahin.ahmadipishkuhi@gmail.com](mailto:mahin.ahmadipishkuhi@gmail.com)

1. **Hojjat Zeraati**

**Affiliation and address**: Department of Epidemiology and Biostatistics, School of Public Health, Tehran University of Medical Sciences, Tehran, Iran, **Email:** [zeraatih@tums.ac.ir](mailto:zeraatih@tums.ac.ir)

1. **Mohammad Ali Mansournia**

**Affiliation and address**: Department of Epidemiology and Biostatistics, School of Public Health, Tehran University of Medical Sciences, Tehran, Iran, **Email:** [mansournia_ma@yahoo.com](mailto:mansournia_ma@yahoo.com)

1. **Azra Ramezankhani**

**Affiliation and address**: Prevention of Metabolic Disorders Research Center, Research Institute for Endocrine Sciences, Shahid Beheshti University of Medical Sciences, Tehran, Iran

**Email:** [ma.ramezankhani@gmail.com](mailto:ma.ramezankhani@gmail.com)

1. **Mahin Ahmadi Pishkuhi**

**Affiliation and address**: Pars Advanced and Minimally Invasive Medical Manners Research Center, Pars Hospital, Iran University of Medical Sciences, Tehran, Iran, **Email:** el.naz66@yahoo.com

1. **Soroush Rostami Gooran**

**Affiliation and address**: Faculty of computer Engineering, Shaid Rajaee Teacher Training University (SRTTU), Tehran, Iran, **Email:** s.rostami@srttu.edu

1. **Akbar Fotouhi**

**Affiliation and address**: Department of Epidemiology and Biostatistics, School of Public Health, Tehran University of Medical Sciences, Tehran, Iran, **Email:** [afotouhi@sina.tums.ac.ir](mailto:afotouhi@sina.tums.ac.ir)

**Corresponding author:** Davood Khalili, Prevention of Metabolic Disorders Research Center, Research Institute for Endocrine Sciences, Shahid Beheshti University of Medical Sciences, Tehran, Iran, Department of Biostatistics and Epidemiology, Research Institute for Endocrine Sciences, Shahid Beheshti University of Medical Sciences, Tehran, Iran

P.O. Box: 19395-4763, Tehran, Iran

Phone: +982122432500

Fax: +982122416264

E-mail:dkhalili@endocrine.ac.ir

**Appendix A**

**Supplementary table 1 :** Description of the classes of lifestyle behaviors in the general population using self-organizing map among the Iranian population in 2011.

|  | | Cluster 1 | Cluster 2 | Cluster 3 | Cluster 4 | Cluster 5 | Cluster 6 | Cluster 7 | Total |
| --- | --- | --- | --- | --- | --- | --- | --- | --- | --- |
| N (percent) | | 1306 (15.84) | 1026  (12.45) | 2781 (33.73) | 566 (6.86) | 1169 (14.18) | 647  (7.85) | 749  (9.09) | 8244  (100) |
|  | | Healthy lifestyle behaviors group | Unhealthy (with high consumption of fast foods, salt, and sweet soft drinks) | Unhealthy (with low physical  activity) | Unhealthy (with smoking and consumption of alcohol and sweet soft drinks) | Unhealthy (without any physical activity and low salt consumption) | Unhealthy (with unhealthy diet, low physical activity, no use of dairy products) | Unhealthy (with considerable smoking and work-related physical activity) |  |
| Nutrition | |  |  |  |  |  |  |  |  |
| Mean servings of fruit in a day | | 1.87 (0.04) | 1.05 (0.04) | 1.37 (0.02) | 1.10 (0.05) | 0.91 (0.03) | 0.38 (0.03) | 0.47 (0.02) | 1.17 (0.06) |
| Mean servings of vegetables in a day | | 1.86 (0.05) | 0.58 (0.03) | 1.07 (0.05) | 0.39 (0.03) | 0.83 (0.07) | 0.35 (0.04) | 0.31 (0.03) | 0.92 (0.04) |
| Mean servings of dairy products in a day | | 2.39 (0.1) | 1.56 (0.08) | 2.03 (0.07) | 0.93 (0.05) | 1.69 (0.08) | 0 | 1.25 (0.06) | 1.65 (0.07) |
| Mean number of days per week eating fast foot | | 0.27 (0.03) | 1.57 (0.05) | 0.01 (0.00) | 0.82 (0.07) | 0.02 (0.00) | 0 | 0.62 (0.05) | 0.38 (0.02) |
| Mean number of days per week drinking sweet soft drinks | | 0.94 (0.04) | 3.15 (0.06) | 0.99 (0.03) | 3.19 (0.10) | 0.78 (0.04) | 1.29 (0.04) | 1.80 (0.07) | 1.64 (0.05) |
| Adding salt to food (percent)* | | 519 (40.74) | 961 (92.66) | 1335 (48) | 345 (62.95) | 439 (37.55) | 250 (37.64) | 489 (68.29) | 4338 (54.46) |
| Predominant use of unsaturated (percent) * | | 372 (28.48) | 435 (42.40) | 1183 (42.54) | 277 (48.94) | 397 (33.96) | 470 (72.64) | 305 (40.72) | 3439 (41.71) |
| Physical activities | |  |  |  |  |  |  |  |  |
| Mean number of hours a week for work related physical activity | | 1.63 (0.12) | 1.68 (0.13) | 0.62 (0.08) | 1.92 (0.09) | 0.24 (0.04) | 1.23 (0.01) | 3.42 (0.21) | 1.49 (0.10) |
| Mean number of hours a week for recreational physical activities | | 1.65 (0.08) | 0.01 (0) | 0 | 0.52 (0.07) | 0.02 (0.01) | 0.02 (0.01) | 0.34 (0.04) | 0.39 (0.03) |
| Mean number of hours a week  for walking | | 0.74 (0.04) | 0.54 (0.04) | 0.35 (0.03) | 0.57 (0.04) | 0.13 (0.02) | 0.43 (0.03) | 0.79 (0.04) | 0.57 (0.02) |
| Tobacco | |  |  |  |  |  |  |  |  |
| Cigarettes (percent) * | | 1 (0.00) | 0 | 0 | 184 (34.51) | 0 | 0 | 749 (100) | 934 (14.01) |
| Hookah (percent) * | | 0 | 0 | 0 | 259 (42.76) | 0 | 0 | 0 | 259 (3.39) |
| Alcohol (percent) * | | 0 | 0 | 0 | 336 (60.29) | 0 | 0 | 0 | 336 (5.52) |
|  |  |  |  |  |  |  |  |  |  |

**Supplementary table 2**: Sociodemographic characteristics of the seven classes of lifestyle behaviors among the Iranian population in 2011.

| Variables name | Cluster 1 | Cluster 2 | Cluster 3 | Cluster 4 | Cluster 5 | Cluster 6 | Cluster 7 | Total |
| --- | --- | --- | --- | --- | --- | --- | --- | --- |
| Age | 37.83 (0.34) | 38.46 (0.47) | 39.16 (0.15) | 39.55 (0.54) | 65.67 (0.07) | 39.13 (0.43) | 43.91 (0.47) | 42.21 (0.03) |
| BMI | 26.62 (0.12) | 27.50 (0.21) | 27.45 (0.11) | 26.16 (0.22) | 27.78 (5.96) | 26.34 (0.22) | 24.63 (0.19) | 26.94 (0.06) |
| Sex (men) | 617 (52.66) | 367 (41.00) | 648 (29.02) | 423 (81.62) | 340 (32.86) | 215 (38.26) | 695 (95.61) | 3305 (46.09) |
| Region (urban) | 1005 (76.67) | 749 (74.82) | 1939 (69.53) | 382 (67.12) | 803 (69.53) | 387 (58.68) | 533 (69.75) | 5798 (69.44) |
| Education |  |  |  |  |  |  |  |  |
| <6 years | 600 (44.51) | 641 (60.50) | 1848 (63.07) | 307 (55.74) | 1070 (88.91) | 509 (73.04) | 562 (71.39) | 5637 (63.57) |
| 6- 12 years | 362 (27.27) | 229 (23.09) | 585 (22.36) | 201 (35.02) | 67 (6.05) | 95 (18.52) | 133 (21.05) | 1572 (22.01) |
| >12 years | 344 (28.22) | 156 (16.32) | 348 (14.57) | 58 (9.24) | 32 (5.04) | 43 (8.44) | 54 (7.56) | 1035 (15.40) |
| Job |  |  |  |  |  |  |  |  |
| Employee working in office | 222 (18.80) | 98 (12.04) | 200 (9.17) | 37 (5.41) | 3 (0.44) | 31 (6.76) | 56 (9.62) | 647 (8.36) |
| Workers in factory environment | 75 (6.30) | 50 (6.79) | 103 (8.14) | 151 (26.56) | 21 (2.56) | 55 (11.96) | 87 (10.56) | 542 (7.36) |
| Self-employed | 241 (19.62) | 195 (22.57) | 379 (16.61) | 234 (40.32) | 125 (17.33) | 107 (19.92) | 352 (46.20) | 1633 (23.31) |
| Housewife | 508 (36.46) | 564 (46.38) | 1764 (51.42) | 38 (7.63) | 771 (47.13) | 379 (48.41) | 38 (8.96) | 4062 (48.01) |
| Others | 260 (18.82) | 119 (12.22) | 335 (14.68) | 106 (20.08) | 249 (32.52) | 75 (12.94) | 216 (24.32) | 1360 (16.12) |
| Age is reported as with weighted mean (SE) and the rest of the variables are reported with unweighted frequency and weighted percent (Data were weighted based on the 2011 national Iranian population aged≥ 25 and ≤ 70 years). | | | | | | | | |
